# Evaluating OCT Device-Reported Image Quality Score: Towards a Task-Specific Quality Gate for Deep Learning-based Outer-Retina and Choroid Boundary Segmentation

**DOI:** 10.64898/2026.05.17.26353399

**Published:** 2026-05-20

**Authors:** Adarsh Gadari, Atharva Ajay Vichare, Francesca Corona, Sharat Chandra Vupparaboina, Shreyaa Rohindra Lall, Giulia Gregori, Nasiq Hasan, José-Alain Sahel, Jay Chhablani, Sandeep Chandra Bollepalli, Kiran Kumar Vupparaboina

## Abstract

Manufacturer-defined signal-strength indices are frequently employed as quality benchmarks for automated optical coherence tomography analysis, yet their empirical relationship with deep learning segmentation accuracy remains unclear. Because these metrics were originally developed for conventional image-processing pipelines, their ability to predict modern model-based segmentation accuracy has not been empirically validated. To address this gap, we evaluated the Heidelberg Spectralis Q-score against U-Net segmentation performance across 5,047 B-scans from 103 eyes for three anatomical boundaries of the posterior segment of the eye: the Ellipsoid Zone (EZ), Bruch’s Membrane (BM), and Choroid Outer Boundary (COB). Alongside standard boundary agreement metrics (MAE, MSE, Dice Similarity Coefficient), we adapted the Earth Mover’s Distance (EMD) from optimal transport theory as a boundary evaluation metric. Unlike column-wise averages, EMD quantifies boundary agreement as a 2-D geometric displacement, directly measuring residual spatial displacement between the model segmented boundary and the ground-truth boundary. Our results demonstrate that the Q-score — originally designed to gate image-processing-based automated analysis — is a poor predictor of deep learning boundary segmentation accuracy, with explained variance ( *R* ^2^ ) failing to exceed 1.4% across all three boundaries. We further observed a monotonically increasing error hierarchy with anatomical depth (EZ < BM < COB), consistent across metrics, which is unexplained by the signal strength. At the COB, correlations were paradoxically positive, explained by a B-scan-level mediation chain: higher Q-scores correspond to greater choroidal thickness ( *r* = 0.113, *ρ* = 0.158), which in turn predicts higher COB segmentation error ( *r* = 0.165, *ρ* = 0.191) — a localization difficulty that global signal strength cannot capture. Collectively, these findings challenge the implicit assumption that signal-strength-based quality thresholds are a reliable proxy for deep learning model performance, and motivate a shift toward task-specific acquisition quality criteria calibrated to model performance rather than signal interpretability.

## Full Text Availability

The license terms selected by the author(s) for this preprint version do not permit archiving in PMC. The full text is available from the preprint server.

